# Uptake and Short-Term Retention in HIV Treatment Among Men in South Africa: The Coach Mpilo Pilot Project

**DOI:** 10.9745/GHSP-D-21-00498

**Published:** 2022-02-28

**Authors:** Mbuzeleni Hlongwa, Morna Cornell, Shawn Malone, Paris Pitsillides, Kristen Little, Nina Hasen

**Affiliations:** a Population Services International, Johannesburg, South Africa.; b School of Nursing and Public Health Medicine, University of KwaZulu-Natal, Durban, South Africa.; c Burden of Disease Research Unit, South African Medical Research Council, Cape Town, South Africa.; d School of Public Health and Family Medicine, University of Cape Town, Cape Town, South Africa.; e Matchboxology, Johannesburg, South Africa.; f Population Services International, Washington, DC, USA.

## Abstract

**Introduction:**

Gender disparities persist across the HIV care continuum in sub-Saharan Africa. Men are tested, linked, and retained at lower rates than women. Men experience more treatment interruptions, resulting in higher rates of virological failure and increased mortality. Peer support is an approach to improving men’s engagement and retention in HIV treatment. We assessed uptake and early retention in HIV care among men in the ‘Coach Mpilo’ peer support pilot project in South Africa.

**Methods:**

We conducted a pilot project from March 2020 to September 2020 in 3 districts: Ehlanzeni and Gert Sibande (Mpumalanga) and Ugu (KwaZulu-Natal). Men living with HIV were invited to receive one-on-one coaching from a peer supporter who was stable on treatment. We analyzed participants’ self-reported data on demographics, uptake, and retention in HIV treatment. We described baseline characteristics using summary statistics and reported uptake and early retention proportions overall and by testing history (newly and previously diagnosed).

**Results:**

Among 4,182 men living with HIV, most were previously diagnosed (n=2,461, 64%) and uptake was high (92%, n=3,848). Short-term retention was 80% (n=1,979) among men previously diagnosed and 88% (n=1,213) among newly diagnosed. In September 2020, 95% (n=3,653/3,848) of all participants reported being active on HIV treatment, including those retained consistently and those who had interrupted and returned to care. Among participants experiencing treatment interruption after enrolling, the majority (82%, n=464) returned to treatment, largely within 2 months.

**Conclusions:**

Improving linkage to and retention in HIV treatment among men is essential for their health and for treatment as prevention. This pilot project provided preliminary evidence that a peer-led support model was acceptable, retained a high proportion of men in the early stages of ART, and supported men returning to care after treatment interruption. These promising results require further investigation to assess impact, scalability, and cost-effectiveness.

## Abstract (precis)

In this pilot project, providing peer support to men living with HIV retained a high proportion of men living with HIV in the early stages of HIV treatment and successfully supported men in returning to care after a treatment interruption.

## Teaser

**Key Findings:**

- Uptake of the Coach Mpilo intervention was high at both the community level and in health care facilities among newly and previously diagnosed men, which was surprising, given the well-documented barriers to men HIV testing and linking to care in sub-Saharan Africa.
- Short-term retention in HIV treatment was high in newly and previously diagnosed men and increased by approximately 20%.
- Of the 16% of men who had at least 1 treatment interruption, almost all men (95%) subsequently returned to antiretroviral therapy within 2 months.

**Key Implications:**

- This study provides important new information on men returning to care after treatment interruption, about whom little is known.
- This model has the potential to be scalable and sustainable because coaches can be recruited from the community, quickly trained, and do not require major infrastructural or operational support from clinics.

## INTRODUCTION

Gender disparities persist across the HIV care continuum in sub-Saharan Africa (SSA).^1^ The rates at which men are tested, initiated, and retained in HIV treatment are lower than for women,^2^ contributing to the growing life expectancy gap between men and women.^2^ In South Africa, 78% of men and 89% of women living with HIV knew their HIV status and 67% of men versus 72% of women diagnosed with HIV were on antiretroviral therapy (ART) in 2017.^3^ Men also experience more frequent treatment interruptions than women,^4^ contributing to increased virological failure^5^ and higher HIV-related mortality rates.^6^ Compared with women, men are 27% more likely to die from HIV, and more than half of all male HIV-related deaths occur in men who have never initiated ART.^7^

To improve men’s health outcomes, curb HIV transmission, and achieve epidemic control, men living with HIV must be linked early and retained in HIV treatment. However, linking and retention remain a serious challenge for ART programs in SSA.^8^ Through formative research, we identified a strong desire among men living with HIV for peer support.

Linking to treatment and retention remain a serious challenge for ART programs in sub-Saharan Africa.

In this study, we assessed the pilot Coach Mpilo peer-support project led by coaches, men who are living openly with HIV, healthy and stable on ART, and have received training and supervision on providing peer support. We assessed uptake of the project and its impact on short-term retention in HIV treatment in 2 groups of men living with HIV in South Africa: men newly diagnosed with HIV and not yet, or recently, initiated onto ART, and men who were previously diagnosed and had experienced a treatment interruption or required adherence support.

## METHODS

### Study Setting

This study was conducted in 3 districts: Ehlanzeni and Gert Sibande in the Mpumalanga province and Ugu in the KwaZulu-Natal province, selected in consultation with provincial and district departments of health. Overall, HIV prevalence is high in these districts, ranging from 20% in Ehlanzeni, 23% in Gert Sibande, and 27% in Ugu.^9^ Among men aged 15–49 years, HIV prevalence is 20% in Ehlanzeni, 21% in Ugu, and 22% in Gert Sibande. Average ART coverage is estimated at 65% in both provinces.^10^ The population is predominantly poor and rural and uses public health services.^11^ The unemployment rate in both provinces was more than 30% in the last quarter of 2020.^12^

### Study Design

This was a pilot project conducted over 7 months (March 2020–September 2020). Men living with HIV who had been stable on treatment for at least 12 months, resided in the study area, and were comfortable disclosing their HIV status were recruited to provide peer support to newly diagnosed men and men experiencing treatment interruption or adherence barriers. Using a sports analogy, coaches were responsible for recruiting and supporting a “team” of “players” (other men living with HIV and interested in receiving peer support) from enrollment in the pilot until September 2020. While formal targets were not set, coaches were encouraged to recruit and support at least 20 men per month.

Coach Mpilo is a variation on the peer mentor or case manager model, employing men living with HIV who have overcome their own barriers and become stable on HIV treatment as coaches of newly diagnosed men and other men struggling to reach treatment stability. The model addresses several of the barriers and needs identified by men during the pilot design phase, breaking the isolation and paralysis that many men feel at diagnosis, giving men a safe and relatable source of support, and giving men living proof that a man with HIV can live a normal life.

Coach Mpilo is a variation on the peer mentor model, employing men living with HIV who have overcome their own barriers and become stable on HIV treatment as coaches of newly diagnosed men and other men struggling to reach treatment stability.

We recruited coaches within their communities and conducted a 1-week training. The customized curriculum draws on accredited HIV counseling and testing content, the BEST (Building, Enhancing, Sustaining, Transitioning) relationships model and the GROW (Goal, Reality, Obstacles [or Options], Way Forward [or Will]) problem-solving model. Training included interpersonal communication, mentorship, establishing and maintaining trust, and building coping and problem-solving skills.

We then linked coaches to a clinic, where they received referrals of men newly diagnosed with HIV and other men in need of support. Coaches also reached out to men proactively using rosters of individuals who had missed appointments and were lost to follow-up as well as doing broader community outreach. Coaches then provided one-on-one mentoring and support tailored to address any psychological, social, or practical barriers to treatment that men were experiencing. This one-on-one support enabled coaches to understand each man’s particular barriers and challenges, build trust and rapport, and provide relevant support, drawing on the coach’s personal experience of living with HIV.

Coaches reported to squad managers, men who had similar characteristics and at least 3 years of experience working with men in communities. Squad managers (supervisors) were responsible for managing and supporting a network of coaches. They monitored how many men their coaches were mentoring and received feedback on the typical challenges facing the participants (players). Squad managers addressed such challenges by providing support, insights, and advice to coaches in the field. Coaches reported to their squad managers weekly. Once a month, squad managers met to share their learnings and experiences. Relevant insights, advice, or mentoring techniques were subsequently fed back to their coaches. Coaches were expected to have at least 1 interaction with each player per week, with the option of more as needed. Coaches received a salary of ZAR3500 (∼US$250) per month and a stipend of ZAR800 ($58) for airtime, data, and transport. Players received no financial incentive to participate.

### Population and Recruitment

Men aged 18 years and older residing in the study areas were eligible for inclusion in the pilot. Participants included those who were newly diagnosed with HIV and either not yet, or recently, initiated onto ART, and men who had been previously diagnosed and had experienced a treatment interruption or required adherence support. Participants were recruited in 2 ways: either contacted directly by a coach during community outreach activities or referred to a coach by a health care facility. The coach or health care provider recorded potential participants’ details into a register. Coaches collected copies of referral registers and enrolled consenting men.

### Definitions

We defined uptake as the proportion of men who consented to participate in the intervention, among all those approached. We defined short-term retention in care as the proportion of participants not reported as interrupting treatment, dead, or transferred out, between enrollment in Coach Mpilo and the project’s end date. We defined treatment interruption as not being on ART for at least 1 month during the pilot period.

### Data Collection and Management

Players reported their treatment status to coaches each month. Squad managers collated data from coaches on a master spreadsheet and submitted this to the research advisor, who audited and imported the data for analysis. We assigned study participants an outcome at the end of the pilot as follows: (1) if the participant was recorded as dead or transferred out, this outcome was assigned; (2) if a participant was recorded as being off treatment or having an unverified treatment status, he was defined as having interrupted treatment; (3) lastly, a participant was defined as retained if he had none of these outcomes and was recorded as being on treatment in September 2020 (last month of follow-up). The day of treatment status in the month of follow-up was not recorded, therefore we assigned the last day of the month as a proxy date. Data were de-identified before final analysis.

### Data Analysis

We entered data into a Microsoft Excel spreadsheet, exported to Stata version 15.0 (Statacorp) for analysis, and then eliminated discrepancies and removed duplicates. We described baseline characteristics using summary statistics (median, interquartile range [IQR], and proportions) overall and by participant group (newly diagnosed versus previously diagnosed). We assessed study participants’ outcomes and reported proportions overall and by participant group. We also described the number of study participants on treatment in September overall and by participant group. Among those who experienced treatment interruption after enrolling in the pilot, we explored outcomes after interruption. We excluded participants who interrupted treatment in September as we lacked sufficient follow-up time to assess their subsequent outcomes. For those who did return to care, we calculated the mean and median time based on assigned proxy dates.

### Ethical Considerations

We obtained ethical approval from the Foundation for Professional Development Research Ethics Committee, the Population Services International Research Ethics Board, and the National Health Research Database. Permissions were obtained from participating provincial departments of health, districts, and facilities before implementation. We obtained written informed consent from all participants to participate in Coach Mpilo. Staff members with access to data were trained on research ethics and signed confidentiality agreements. To ensure confidentiality, we used project-specific unique identifying codes stripped of personal identifying information.

## RESULTS AND DISCUSSION

The pilot included 3,848 males from 73 clinics, with approximately one-third newly diagnosed (n=1,387, 36%) and two-thirds previously diagnosed with HIV (n=2,461; 64%) (Table 1). Characteristics at enrollment were similar in both groups. The median age was 35 years (IQR: 30–42), and almost half (n=1,792; 47%) were aged 30–39 years. Enrollment varied by district, with Gert Sibande accounting for half of all participants (n=1,909; 50%) and having a relatively higher proportion of newly diagnosed men (43% versus 36% in Ehlanzeni and 21% in Ugu). Among participants who initiated ART before enrollment in the pilot, median time on ART was 1 month among newly diagnosed men and 42 months among previously diagnosed men.

**TABLE 1.** Characteristics of Men in the Coach Mpilo Pilot Project, KwaZulu-Natal and Mpumalanga Provinces, South Africa, 2020

	**Newly Diagnosed,** **n=1,387 (36%)** **No. (%)**	**Previously Diagnosed,** **n=2,461 (64%)** **No. (%)**	**Total** **N=3,848**
Age, median (IQR), years	34 (29–39)	36 (30–43)	35 (30–42)
Age range, years			
15–19	27 (1.9)	62 (2.5)	89 (2.3)
20–29	327 (23.6)	436 (17.7)	763 (19.8)
30–39	687 (49.5)	1105 (44.9)	1792 (46.6)
40 and older	346 (24.9)	858 (34.9)	1204 (31.3)
Racial group			
African	1381 (99.6)	2452 (99.6)	3833 (99.6)
Asian	6 (0.4)	9 (0.4)	15 (0.4)
Distribution by district			
Gert Sibande	838 (60.1)	1071 (43.5)	1909 (49.6)
Ugu	226 (16.3)	826 (33.6)	1052 (27.3)
Ehlanzeni	323 (23.3)	564 (22.9)	887 (23.1)
Time on ART at recruitment, months^a^	275	769	1,044
Median (IQR)	1 (1–3)	42 (18–65)	24 (5–57)

Abbreviations: ART, antiretroviral therapy; IQR, interquartile range.

a Restricted to participants who initiated ART before enrollment to Coach Mpilo.

### Uptake of the Coach Mpilo Intervention

Uptake of the Coach Mpilo intervention was high: among 4,182 men living with HIV and invited to participate, 92% (N=3,848) gave consent to be enrolled in the program, with similar uptake in all districts. Such high uptake was surprising, given the well-documented barriers to men testing and linking to HIV care in SSA.^13^^,^^14^ In South Africa, for example, compared with women, men have 4 times higher odds of not being tested for HIV; and men living with HIV have nearly twice the odds of not knowing their HIV status compared with women.^15^ Consequently, men generally initiate ART when they are older and sicker than women,^16^ resulting in higher morbidity and mortality.^17^

Such high uptake in the Coach Mpilo project was surprising, given the well-documented barriers to men testing and linking to HIV care in SSA.

In recent years, there is growing awareness of the critical need for effective strategies to increase HIV testing and earlier linkage to care for men in SSA.^18^ Some successes have been reported in community-based compared with facility-based HIV testing and ART initiation.^19^^–^^21^ However, the coronavirus disease (COVID-19) pandemic has severely reduced HIV testing and ART initiation in many countries. For example, South Africa reported a 47.6% and 46% decrease in HIV testing and ART initiation, respectively, in April 2020, the first month of lockdown.^22^ Despite this, we found high uptake not only at the community level, reaching men generally regarded as hard to reach, but also in health care facility settings. Newly diagnosed as well as previously diagnosed men were keen to participate in this peer support-based model.

### Short-Term Retention in HIV Treatment

Short-term retention (with no treatment interruptions) was high in both groups, ranging from 80% (n=1,979) among men previously diagnosed to 88% (n=1,213) among those newly diagnosed (Table 2). Overall, 618 (16%) participants had at least 1 treatment interruption, with newly diagnosed and previously diagnosed men accounting for 12% (n=168) and 18% (n=450) respectively. Overall, 28 men (0.7%) transferred out and 10 men (0.3%) died.

**TABLE 2.** Short-term Outcomes Among Participants in the Coach Mpilo Pilot Project, KwaZulu-Natal and Mpumalanga Provinces, South Africa, 2020

**Outcome**	**Overall** **No. (%)**	**Newly Diagnosed** **No. (%)**	**Previously Diagnosed** **No. (%)**
Died	10 (0.3)	1 (0.1)	9 (0.4)
Interrupted treatment	618 (16.1)	168 (12.2)	450 (18.3)
Retained	3192 (83.0)	1213 (87.5)	1979 (80.4)
Transferred out	28 (0.7)	5 (0.4)	23 (0.9)

Short-term retention on ART is critical, given the elevated risk of morbidity and mortality within the first few months on treatment and after treatment interruption.^23^ Studies have confirmed poorer retention in HIV care among men than women at each stage of the HIV care continuum in SSA.^24^^–^^27^ Our pilot increased early retention by ±20% compared with background rates for these districts,^28^ providing promising preliminary results that merit further investigation. For example, quarterly cohorts’ retention rates were 66.5% and 66.2%, in Ugu District and KwaZulu-Natal province, respectively.^28^ In Ehlanzeni District, Mpumalanga province, the retention rates were 64% and 69%, respectively, in quarters 1 and 2 of FY2020. Low rates of retention in care have also been reported in other studies conducted in similar settings.^24^^,^^27^

### Outcomes After Treatment Interruption in the Coach Mpilo Pilot Project

Among the 618 participants who experienced at least 1 treatment interruption while enrolled in Coach Mpilo, we excluded 55 (9%) who interrupted treatment in September and lacked follow-up time to assess subsequent outcomes. Among the remaining 563 participants, the majority (82%, n=464) returned to HIV treatment during the pilot period, ranging from 78% (n=118) of newly diagnosed to 84% (n=346) of previously diagnosed men (Figure). As of September 2020, 95% (n=3,653) of all participants reported being active on HIV treatment, including those retained consistently as well as those who had experienced a treatment interruption but returned to care within the pilot period.

**FIGURE:**
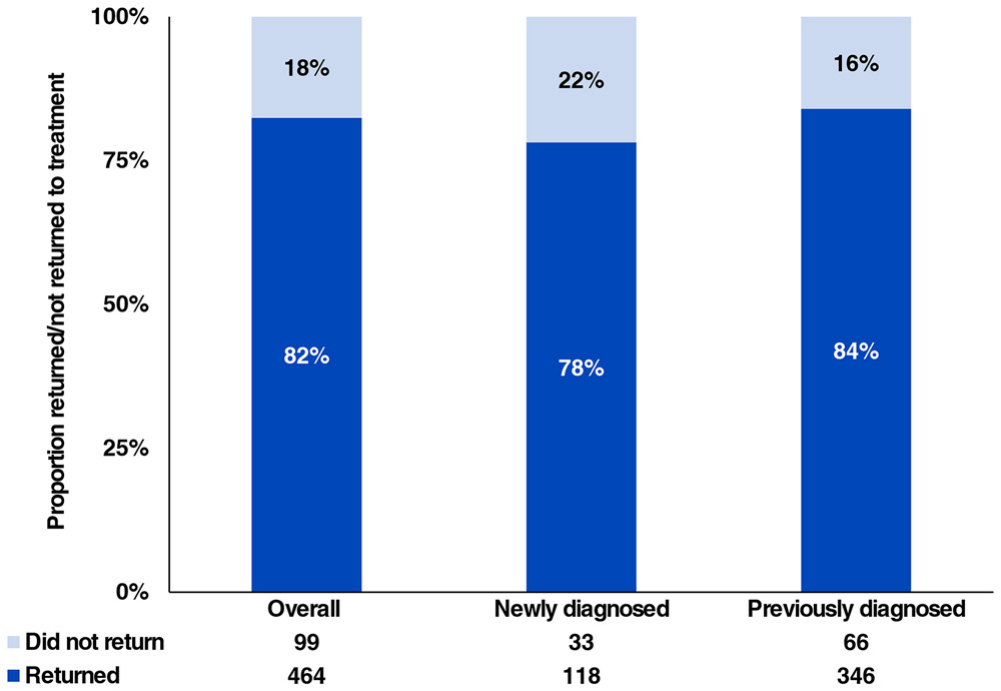
Outcomes of Participants After HIV Treatment Interruption During the Coach Mpilo Pilot Project in 2 Provinces in South Africa, 2020

This study provides important new information on men returning to care after treatment interruption, about whom little is known. Almost all men who experienced treatment interruption subsequently returned to ART within 2 months, conflicting with results of previous studies reporting that men were less likely to resume treatment than women.^29^^–^^32^ In Kenya, 65% of 4,050 individuals who never returned after treatment interruption were male.^30^ In South Africa, only one-third of patients who interrupted care returned to HIV treatment.^29^ The high return rate in our study suggests that the pilot project successfully supported men retained consistently in care as well as those who returned to care after a treatment interruption.

Almost all men who experienced treatment interruption subsequently returned to ART within 2 months, conflicting with results of previous studies reporting that men were less likely to resume treatment than women.

### Scalability and Sustainability

Several factors inform the potential scalability and sustainability of the model. Coaches can be recruited locally in any community where men are living with HIV who are stable on treatment. They can be trained within 1 week, and the training does not rely on any prior knowledge or education. Coaches are compensated at a similar level as other entry-level public health cadres, for example, lay counselors and community health workers. Being community-based, the coaches do not require major infrastructural or operational support from the clinics. Clinic teams and implementing partners found the model feasible and acceptable. Since the pilot was conducted, 7 U.S. President’s Emergency Plan for AIDS Relief partners in South Africa have integrated the model into their large-scale treatment programs.

### Limitations

Our findings are subject to important limitations. We planned to verify treatment status by exporting de-identified patient-level data directly from the TIER.Net database, an electronic patient management system capturing patient-level data on public sector HIV management in South Africa. However, changes in data access policies to protect patient privacy enacted shortly after the pilot’s launch prevented any linkage to TIER.Net. Thus, we relied on self-report, and our results may reflect social desirability bias and overestimate true retention. Future studies should include validation of participants’ treatment status, preferably with measured viral suppression as a proxy for adherence. The project also experienced major disruptions due to COVID-19, including restrictions on physical movement, multimonth dispensing, changes in operating hours, designation of some clinics as COVID-19-only, and general fear of visiting health facilities. In the next phase, we will assess impact, scalability, and cost-effectiveness more robustly, using lessons learned from the pilot.

## CONCLUSION

Improving linkage to and retention in HIV treatment among men is essential for their health and for treatment as prevention. In this pilot project, providing peer support to men living with HIV was acceptable to men, helped retain a high proportion of men living with HIV in the early stages of HIV treatment, and helped support men in returning to care after a treatment interruption. This promising approach merits further investigation to validate impact and assess scalability and cost-effectiveness.

## Notes

**Peer Reviewed**

**First published online:** February 11, 2022.
